# Feasibility Study on the Application of Biodegradable Plastic Film in Farmland Soil in Southern Xinjiang, China—Planting Tomatoes as an Example

**DOI:** 10.3390/toxics11050467

**Published:** 2023-05-18

**Authors:** Rehemanjiang Wufuer, Jia Duo, Liang Pei, Shuzhi Wang, Wenfeng Li

**Affiliations:** 1 State Key Laboratory of Desert and Oasis Ecology, Xinjiang Institute of Ecology and Geography, Chinese Academy of Sciences, Urumqi 830011, China; reheman319@ms.xjb.ac.cn (R.W.); duojia2017@ms.xjb.ac.cn (J.D.); peiliang@igsnrr.ac.cn (L.P.); wangshuzhi@ms.xjb.ac.cn (S.W.); 2 Xinjiang Key Laboratory of Environmental Pollution and Bioremediation, Xinjiang Institute of Ecology and Geography, Chinese Academy of Sciences, Urumqi 830011, China

**Keywords:** induction period, mulching, degradation rate, soil humidity

## Abstract

In recent years, polybutylene adipate-co-terephthalate (PBAT) mulch film has become one of the most commonly used biodegradable mulch films in agriculture in an attempt to combat plastic film pollution. However, its degradation characteristics and impact on the soil environment and crop growth are affected by many factors such as its composition, soil and crop types, local climate characteristics, etc. In this study, PBAT mulch film and ordinary polyethylene (PE) film were used as test materials, with non-mulching treatment (CK) as a control, to study the applicability of PBAT film in Moyu County, Southern Xinjiang region, using tomato growth as an example. The results showed that PBAT film started its induction period after 60 days, and 60.98% of the PBAT film was degraded within 100 days. Generally, the soil temperature and humidity preservation functions of this film were comparable to that of PE film in the seedling and flowering–fruiting stages of tomato growth. In the mature stage, the soil humidity under PBAT film was significantly lower than that of PE film due to its substantial degradation rate. However, this did not have any significant negative effects on tomato growth, yield, and quality. The tomato yield of 667 m^2^ with BPAT was insignificantly lower than that of PE film by 3.14%, and both were significantly higher than that of the CK treatment by 63.38% and 68.68%, respectively, indicating that it is feasible to use PBAT film to cultivate crops such as tomato in the arid region of Southern Xinjiang, China.

## 1. Introduction

South Xinjiang belongs to a typical continental arid climate, characterized by long sunshine, large temperature difference between day and night, drought, and little rain with annual evaporation of more than 2300 mm, a frost-free period of about 240 days, and annual precipitation of only 20–100 mm [1]. South Xinjiang is an important agricultural production base in China, producing cotton, corn, fruits, and vegetables. Most crops, especially cotton, are covered with plastic film for growth [2]. In 2017, the total area covered with plastic film in Xinjiang was 3.7978 million hm^2^, and the amount of plastic film used was 0.219 million tons [3], accounting for 14.6% of the total amount of plastic film used in China. The extensive use of agricultural mulching film has made the white pollution in Southern Xinjiang worse every year. In 2016, the average amount of farmland residual film in the whole of Xinjiang was 121.5 kg ha^−1^, ranging from 0 to 502.2 kg ha^−1^ [4]. Currently, most agricultural mulching films are made of polyethylene materials, which have a stable chemical structure and are difficult to degrade [5]. The mulch film left in the farmland affects soil structure and function, reduces soil fertility and the germination rate of the seeds, further affects the quality of subsequent crops, and seriously hinders the sustainable development of the agricultural industry [6].

In recent years, biodegradable plastic films including polylactic acid (PLA), polycaprolactone (PCL), polybutylene succinate (PBS), PBAT, and their mixtures have been widely used in agriculture in an attempt to reduce plastic film pollution [5]. These polymers can be used by microorganisms in the soil and decomposed into carbon dioxide and water. Among them, PBAT mulch film has been of great interest due to its good ductility, elongation at break, heat resistance, and impact resistance [6,7]. The annual production of PBAT mulching film increased from 11,900 tons in 2011 to 33,800 tons in 2018, accounting for 7.2% of the total output of bioplastics worldwide [8,9]. PBAT mulch film has been trialed in the production of crops such as cotton [10,11,12,13,14], corn [15,16,17,18], tomato [19,20], pepper [11], potato [21], and beet [22]. These research results showed that PBAT film is capable of fulfilling the main functions of PE film such as heat preservation, moisture conservation, crop growth, and yield promotion. However, the degradation characteristics of PBAT film and its effects on crop growth and yield are varied depending on different crop types, film mulching methods, irrigation amounts, and regional climatic conditions [23,24,25]. Currently, there are few studies on the application of PBAT mulch in South Xinjiang’s arid regions, especially in the Hetian region, and its degradation characteristics and effects on crops are unknown.

Moyu County is located in the northeast part of the Hetian region, at the northern foot of the Kunlun Mountains and the southern edge of the Taklimahan Desert. The region has a warm, arid desert climate with an average altitude of over 1300 m. The annual average temperature is about 9.4 °C with a short frost-free period and long sunshine time. The annual precipitation is 36 mm and annual evaporation is up to 2480 mm. In 2020, the total area of cultivated land in Moyu County was 37352 ha, in which cotton and facility agriculture fields (about 5670 ha) mainly use traditional PE plastic film [26]. Therefore, it is necessary to assess the feasibility of using biodegradable mulch film PBAT and to study its degradation properties and impact on soil temperature and humidity, tomato growth, and yield in order to assess its applicability in this region. The relevant outcomes could provide a scientific basis for curbing plastic film pollution and promoting biodegradable mulch film in the whole Southern Xinjiang region.

## 2. Materials and Methods

### 2.1. Site Description

This test was conducted in the farmland of Dunarexi Village, Jiahanbage Township, Moyu County, Hotan Prefecture, Xinjiang, from May to August 2020. The coordinates are 37°11.35′ N, 79°43.05′ E. The test area is flat, and the soil type belongs to anthropogenic–alluvial soil. The basic soil properties are given in Table 1.

### 2.2. Used Materials

The used film mulching material was biodegradable plastic film, black, 70 cm wide, and 0.008 mm thick, provided by Xinjiang Lanshan Tunhe Degradable Materials Co., Ltd., (Changji, China) mainly composed of PBAT (polyadipic acid/butylene terephthalate). The ordinary PE film (70 cm wide and 0.008 mm thick) was purchased at the local farmers’ market and mulched on 30 April 2020.

The used tomato variety was Zhongshu No. 4 tomato (Jinshengyuan Seedling Co., Ltd., Xinmin, China), commonly grown in Moyu County. After 25 days of cultivation in the vegetable greenhouse, the seedlings were transplanted to the experimental field on May 1st.

### 2.3. Experimental Design

**Figure 1:**
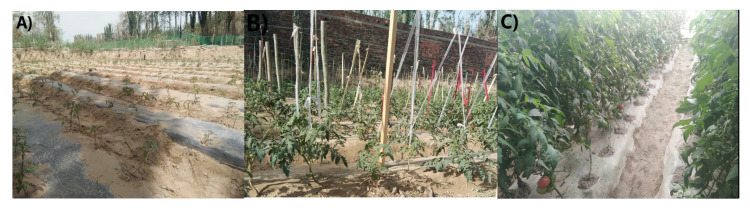
Different stages of tomato growth. (**A**) Seedling stage, (**B**) flowering and fruiting stage, and (**C**) mature stage.

Three treatments were set up in the experiment, including PBAT biodegradable plastic film, PE plastic film, and uncovered land without plastic film (CK), respectively. Each treatment was repeated three times (a total of 9 plots) with a single area of 30.6 m^2^ (34 m × 0.9 m). Each plot had a random block arrangement and ridge planting in the field. The width of the ridge, the row spacing, and the transplanting hole spacing were 50 cm, 40 cm, and 40 cm, respectively. Before ridge planting, 50 kg of ternary compound fertilizer (N 15%, P 4.3%, K 8.3%) was used. The plastic film was manually overlaid on the ridge surface, and the two sides of the plastic film were buried on either side of the ridge. The tomato seedlings with neat growth were selected for planting. Each ridge was planted in two rows. The surface irrigation method was used roughly once every 15 days. The field management of fertilizer dosage, pest control, and weeding was consistent with the local field regulations. Tomatoes were planted on 1 May 2020, and then harvested on 24 August–14 September 2020. The different growth stages of tomatoes are shown in Figure 1.

### 2.4. Test Methods

#### 2.4.1. Degradation Rate and Intensity of the Plastic Films

After mulching, film photographs were taken at fixed points every 20 days throughout the growth period of tomato. Changes on the film surface, such as cracks and holes, were observed and recorded at the points of observation to determine the film induction period. Next, PBAT and PE plastic films were prepared using rectangular slices of 30 cm and 15 cm in length and width, respectively, weighing 0.61 g/piece and 0.58 g/piece, respectively. A total of 12 slices of each plastic film type were covered on the surface of the test field and taken back to the laboratory periodically for analysis. The slices were gently washed with deionized water, scrubbed with 95% ethanol, washed again with deionized water, and dried at room temperature for 48 h before weighing.

#### 2.4.2. Atmospheric, Soil Temperature, and Humidity

The ambient air temperature and relative humidity near the test farmland were recorded once an hour during the whole growth period using a field air temperature and humidity recorder (EL-USB-2USB, Lascar, Shanghai, China). The soil temperature and humidity were measured and recorded separately at the depth of 0~10 cm and 10~20 cm in the farmland soil at 10:00, 14:00, and 22:00 Beijing time in a typical day of different growth stages using a portable soil moisture temperature conductivity meter (TDR150, Beijing, China). The stages of tomato growth include the seedling stage, the flowering–fruiting stage, and the mature stage.

#### 2.4.3. Plant Height and Stem Diameter of Tomatoes

At each growth stage, three representative tomato plants with normal growth were selected and marked in each plot, and their plant height and stem diameter were regularly measured and recorded. Plant height is the natural height from the root base of the plant to the top growth point, measured with a ruler. The thickness of the stem is 1 cm below the first true leaf and measured with a Vernier caliper. The daily average plant height and stem diameter growth were calculated by dividing by the overall growth period, starting from the early stage of the tomato plant.

#### 2.4.4. Yield and Vegetable Quality

##### Yield

A sample of 5 consecutive tomato plants with even growth was selected for each plant’s yield from the different treatments, and the number and weight of the vegetables were counted and measured. Mean values were calculated by dividing by the number of plants chosen. The plot yield was the average of the three plot yields corresponding to each treatment. The yield of 667 m^2^ for each treatment was calculated according to the plot area and plot yield.

##### Quality

The main quality parameters of tomatoes including vitamin C (VC), soluble sugar, and soluble protein were determined by selecting 5 tomatoes with moderate maturity from each treatment. The contents of VC, soluble sugar, and soluble protein were determined using the ultraviolet fast method, sulfuric acid–anthrone method, and micro-Kjeldahl method, respectively.

#### 2.4.5. SEM Analysis

**Figure 2:**
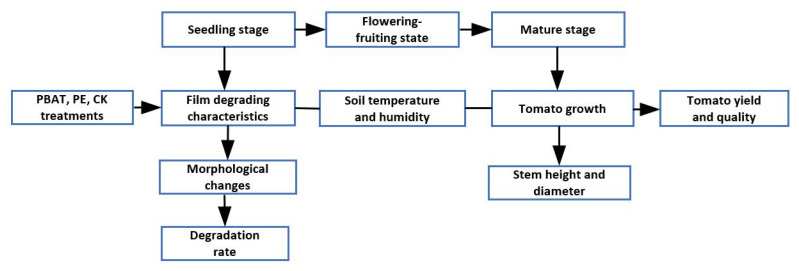
Flowchart showing the treatments and measurements.

The PBAT and PE films were soaked in 75% ethanol for 0.5 h, rinsed with distilled water 3 times, and put in an air-drying oven for 24 h at 60 °C before SEM analysis. A scanning electron microscope (Zeiss Super 55VP, Oberkochen, Germany) was used for analyzing the morphological changes on the surface of the PBAT and PE film samples. A flowchart showing the treatments and measurements is given in Figure 2.

### 2.5. Statistical Methods

The data analysis was performed using Microsoft Office 2016 (Microsoft, Redmond, OR, USA) and OriginPro 9.1 (Originlab, Northampton, MA, USA). The ANOVA statistical method was applied to determine whether there were significant differences in the mean values of this study. The significance level for the analysis was set at *p* < 0.05.

## 3. Results

### 3.1. Degradation Characteristics of Plastic Film in Different Periods

#### 3.1.1. Changes in Surface Morphology of Plastic Film

**Figure 3:**
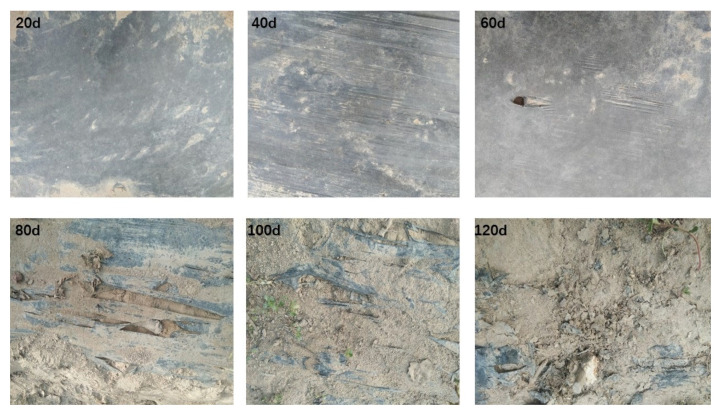
Changes in PBAT plastic film surface morphology during tomato growth; d means days.

As shown in Figure 3, there is a significant gradual degradation trend in the PBAT film with the growth process of tomatoes in general. After 20 and 40 days of field coverage, the surface of the PBAT film did not show any significant cracks, but the surface was observed to become thinner. After 60 days, cracks began to appear, and the surface further thinned, indicating that the PBAT film started its inductive period. After 80 days of coverage, a large number of reticular fissures appeared, and the PBAT film entered a rupture phase. After 100 days, the cracks expanded further and the PBAT film became brittle and adhered to the ground. After 120 days, no significant large PBAT film remained on the ground, and 60% of the ridge was free of any visible PBAT film. In contrast, the PE mulch film showed no cracks in a wide range of areas, except for individual damages caused by agricultural operations, indicating that there was no significant degradation in the PE film during the entire growth period.

#### 3.1.2. Changes in the Microstructure of Plastic Film

**Figure 4:**
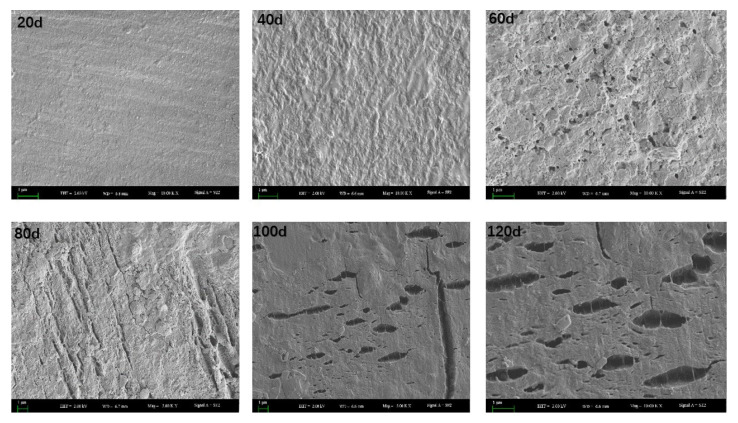
Microscopic morphological changes in PBAT mulch film during tomato growth; d means days.

Figure 4 shows the microscopic morphological changes in the PBAT film after degradation at different stages. It can be seen from the SEM photos that the PBAT film surface was still intact and smooth without any cracks after 20 days of coverage. After 40 days of coverage, irregular bumps and depressions appeared on the surface, but the surface remained intact without any holes or cracks. After 60 days, a large number of small holes and cracks appeared on the surface of the PBAT film, indicating the induction period of the PBAT film. After 80 days, many strip-like cracks appeared on the surface, which further expanded after 120 days, hinting at an acceleration in the degradation process. It is also obvious from Figure 4 that the degradation process on the film surface was uneven, which could be attributed to the inconsistent degradation rate of BA and BT components in the PBAT film structure [27]. In contrast, the surface morphology of the PE mulch did not change significantly, which is consistent with surface observations of PE mulch, indicating that the PE mulch was not degraded significantly during the entire growth period.

#### 3.1.3. Changes in Weight Loss and the Degradation Rate

The initial weight of PBAT and PE plastic films were 0.62 g/piece and 0.58 g/piece, respectively. The weight loss and degradation rate at different time intervals are given in Table 2.

As can be seen from Table 2, the weight loss increased with time for both PE and PBAT films at different rates. Only 2.13% and 5.73% of the PBAT film lost weight after 20 and 40 days, respectively, and this increased dramatically to 15.05% after 60 days, which also indicates that the PBAT film started its induction period during this time. The film loss rate reached 60.98% after 100 days of coverage, and increased by about 3 times after 40 days, indicating that the biodegradable plastic film entered an accelerated rupture phase during this period. However, the overall PE film weight loss rate during this period was only 1.47%, which was insignificant to the original weight (*p* > 0.05).

### 3.2. Effect of Different Mulching Treatments on Soil Temperature and Humidity

#### 3.2.1. Effect of Different Covering Treatments on Soil Temperature

**Figure 5:**
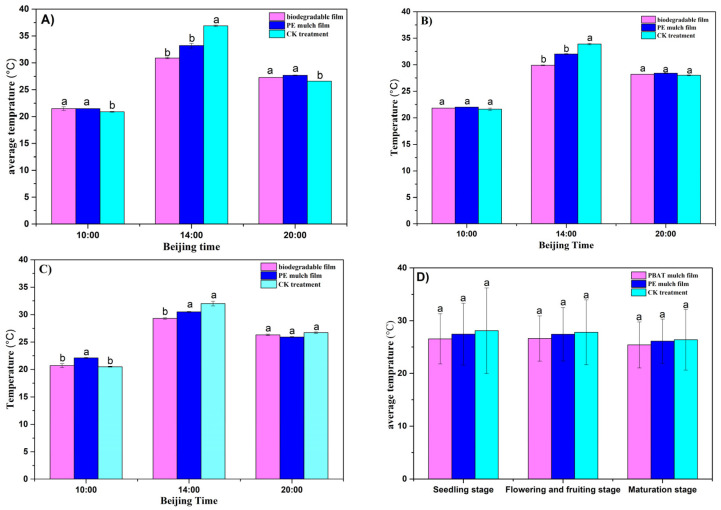
Change in soil temperature at different growth stages and different time periods. (**A**) Seedling stage, (**B**) flowering and fruiting stage, (**C**) mature stage, and (**D**) daily average temperature. The a and b symbols indicate the significance of the difference.

Figure 5 shows the daily variation trend in the average soil temperature from 0 to 20 cm for different treatments during tomato growth. Figure 5A shows that at 10:00 in the tomato seedling stage, the average temperature of the soil covered with PBAT film was equivalent to that covered with PE plastic film, both of which were 0.6 °C higher than that of the CK treatment with a significant difference (*p* < 0.05). At 20:00, the average soil temperature covered with PBAT film was 0.4 °C lower than that covered with PE plastic film without a significant difference, and 0.7 °C higher than that in the CK treatment with a significant difference (*p* < 0.05). However, at 14:00, the temperature increase in the CK treatment was significantly higher than both PBAT and PE films (*p* < 0.05) due to the hot temperature at noon from May to August. This illustrates that both PBAT and PE films played a role not only in maintaining the soil temperature but also in delaying and regulating the soil temperature during hot periods. At 10:00 and 20:00 in the flowering–fruiting stage, the soil temperature covered with PBAT film was 0.2 °C lower than that covered with PE plastic film, and 0.2 °C higher than that of the CK treatment, with no significant differences (see Figure 5B). At 10:00 in the maturing stage, the average soil temperature covered with PE mulching film was 1.4℃ and 1.6 °C higher than that covered with PBAT film and in the CK treatment with significant differences, while that covered with PBAT film was 0.2 °C higher than that of CK without a significant difference. This is because the PBAT film has already started its rupture phase and essentially lost its soil temperature preservation function at this stage. At 20:00, there was not a significant difference in soil temperature between the three treatments, as shown in Figure 5C. Figure 5D depicts the daily variability in the average soil temperature at different tomato growth stages. As seen in Figure 5D, at different stages of tomato growth, the daily average soil temperature in three treatments was in the order of CK treatment > PE film > PBAT film without significant differences (*p* < 0.05). This is mainly due to the fact that the entire growing period for tomatoes was from May to August, and the soil temperature was particularly affected by strong solar radiation during this period. Overall, the effect of film mulch on soil temperature in our study was not significant due to the high temperature during tomato growth, implying that the effect of film mulching cannot be assessed simply by whether there is a temperature increase.

#### 3.2.2. Effect of Different Treatments on Soil Humidity

**Figure 6:**
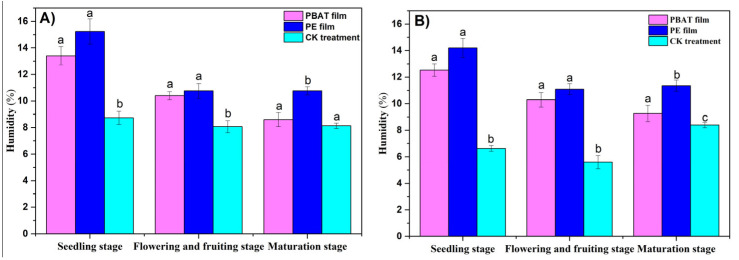
Changes in soil humidity at different depths for the different growth stages (%). (**A**): 0~10 cm, (**B**): 10~20 cm. The a–c symbols indicate the significance of the difference.

Moyu County is located in the northwestern part of the Hetian region and the southern edge of the Taklimakan Desert, with a low rainfall and dry climate. The main role of film mulching is reflected in its moisturizing capacity. It can be seen from Figure 6A that the soil humidity (at 0~10 cm) covered with PBAT film in the seedling stage and the flowering–fruiting stage was 13.40% and 10.40%, respectively, which was 1.92% and 0.37% lower than that covered with PE plastic film without significant differences (*p* > 0.05). The soil humidity for both PBAT and PE films at this depth was significantly higher than that in the CK treatment (*p* < 0.05), indicating that the PBAT film had the same effect as the PE film in keeping the soil humidity during these two stages of tomato growth. However, during the mature stage, the humidity of the soil covered with PBAT film at 0~10 cm was 8.60%, which was 2.17% lower than that covered with ordinary PE film with a significant difference (*p* < 0.05), and 0.47% higher than that in the CK treatment without a significant difference (*p* > 0.05). This can be explained by the fact that the PBAT membrane retained a good moisture retention performance during the seedling and flowering stages before the induction period, and gradually lost its moisture retention performance due to the induced cracking during the mature state. The change in soil humidity at 10~20 cm showed a similar trend to that at 0~10 cm (see Figure 6B). At the seedling and flowering–fruiting stages, the humidity at 10~20 cm for the soil covered with PBAT film was slightly lower than that covered with PE film (*p* > 0.05) and significantly higher than that in the CK treatment (*p* < 0.05). During maturation, the moisture content of the soil covered with the PBAT film at this depth was significantly lower than that covered with the ordinary PE film, and there was no significant difference with respect to the CK treatment.

### 3.3. Effects of Different Treatments on Tomato Growth, Yield, and Quality

#### 3.3.1. Effects on Tomato Growth

Table 3 shows the changes in plant height and stem diameter for tomatoes within the three treatments at different growth stages. As seen in Table 3, the plant height and stem diameter of tomatoes gradually increased with the growth period of the tomato. The plant height in the three treatments was in the order of PBAT film > PE film > CK treatment in the seedling stage without significant differences. In the flowering–fruiting stage, tomatoes treated with PBAT and PE films were 9.8% and 12.9% taller, respectively, than those treated with CK, with significant differences. The plant height of tomatoes with PBAT film was lower than those with PE film, but the difference was not significant. In the mature stage, the trend in plant height changes is similar to that in the flowering–fruiting phase. The plant height of tomatoes with PBAT film was slightly lower than that with PE film, but the difference was not significant, but both were significantly higher than the CK treatment (14.2% and 17.4% higher than CK, respectively). It can be also found from Table 3 that in the seedling and flowering–fruiting stages, the thickness of tomato stems was in the order of PE film >PBAT film >CK treatment, without significant differences. In the mature stage, there was no significant difference in stem diameter between the PBAT film and PE film, but both were significantly higher than the CK treatment (6.6% and 10.5% higher than CK, respectively), indicating that the PBAT film had the same promotion effect on tomato growth as the PE film, both of which were significantly higher than the CK treatment.

#### 3.3.2. Effect of Different Treatments on Tomato Yield

Table 4 provides the data from the different treatments on tomato yield per plant and yield of 667m^2^. As shown in Table 4, the average yield per plant from tomatoes covered with PBAT and PE plastic film was higher than that of CK by 59.3% and 73.3%, respectively, with significant differences. Meanwhile, the yield per plant of tomato covered with PBAT film was 9.44% lower than that covered with PE film without a significant difference. In terms of average plot productivity, the yield increases (compared to CK) for PBAT film and PE film were 63.38% and 68.68%, respectively, without a significant difference. However, both were significantly higher than that in the CK treatment, indicating that the positive effect of the PBAT film on tomato yield was basically equivalent to that of the PE film.

#### 3.3.3. Effect of Different Plastic Films on Tomato Quality

Table 5 shows the content of VC (vitamin C), soluble sugars, and soluble protein in tomatoes with different treatments. As seen in Table 5, the tomato VC content was in the order of CK treatment > PE film > PBAT film, while the soluble sugar content was CK treatment > PBAT film > PE film, and the soluble protein content was PBAT film > PE film > CK treatment. Generally, there were not significant differences between each nutrient group in three treatments, implying that there is not a correlation between tomato yield and quality. Therefore, the effect of PBAT film mulching on tomato quality was insignificant.

## 4. Discussion

The development of degradable mulching film has provided an efficient means to alleviate white pollution in agriculture. However, selecting the right type of degradable plastic film with an appropriate degradation rate and lifetime is essential to ensure high crop yield and reduce agricultural residual plastic film pollution [28]. In our study, the PBAT film started its induction period by exhibiting some crackling after 60 days, and about 60% of the ridge was rid of the film through an accelerated degradation process before the harvest period. The degradation degree and speed of degradable mulch film vary due to differences in raw material composition, production and processing technology, and growing environments [29]. For instance, Wu Si et al. [30] showed that PBAT plastic film developed small cracks after 180 days under indoor conditions, and the degradation rate was related to soil moisture content. Wang Long et al. [10] and Dilibaier Dilimaitai et al. [31] reported that the induction period for three kinds of PBAT mulches with different contents was between 50 and 62 days and 58 and 62 days, respectively. Except for PBAT film, starch-based and polylactic acid-based biodegradable plastic films were reported to start their induction period after about 20 days and degraded into large fragments after 60 days [32,33]. Another kind of photo-biodegradable film was also shown to have cracks after 30–40 days of mulching, and a large area of cracking occurred after 90 days [34]. The induction period of biodegradable film using PBAT as a raw material was longer than that of the starch and polylactic acid-based biodegradable and photo-biodegradable films and can meet the planting demand of tomatoes in Sothern Xinjiang.

Soil temperature and moisture are very important factors that directly or indirectly affect crop growth, yield, and quality. In this study, the soil temperature under PBAT mulch film at 10:00 p.m. and 20:00 p.m. was significantly (0.6 °C and 0.7 °C, respectively) higher than that in the open field (CK) in the seedling stage, but it was insignificantly higher (both 0.2 °C) in the flowering–fruiting stage and equivalent to that of PE mulch film in both stages. After entering the rupture period, cracks appeared on the PBAT film surface, which led to significantly lower soil temperature than that under the PE film in the mature stage. Ibarra et al. [35] found a positive correlation between crop earliness and soil heat accumulation under mulching treatments and no significant differences between plastic and biodegradable films. Generally, the soil heat-conserving performance of the biodegradable film was better before cracking, which was equivalent to that of the PE film, and consistent with the conclusions of Wang [36] and Shen [34]. The main role of film mulching is reflected in its moisture-keeping performance in arid regions. The average soil humidity under the PABT film was significantly higher (10.57% and 7.03%) than in the open field and not significantly different from the PE film in both the seedling and flowering–fruiting stages. Wang Bin et al. [37] found that the average soil humidity was 2.26% lower than that of PE film during the whole tomato growth process, with an insignificant difference. However, in our study, soil moisture was significantly lower (4.27%) than that under the PE film due to the degradation with cracks in the mature stage. Therefore, the degradation period of degradable plastic film should be appropriate and meet the needs of soil temperature and humidity at different growth stages of crops.

In contrast to the significant increases in soil temperature and humidity in the seedling stage, there were no significant differences in tomato plant height and stem sickness for all three treatments during this period. In flowering–fruiting and mature stages, the plant height under PBAT film was 3.2 cm and 9.88 cm higher than those in the CK treatment with significant increases. The stem thickness of the tomato showed a similar growth trend as the plant height during the whole growth period. In general, tomato growth under the PBAT film was not significantly different from the PE film, which is consistent with other reports on cotton [13,38] and corn [18].

The yield of tomatoes covered with PBAT film was not significantly different than that covered with PE film with a 3.14% decrease, but it was significantly higher than that of the CK treatment by 63.38% in our study. In other studies, the yield of corn [13] treated with PBAT and PE plastic films was increased by 20.9% and 21.6%, respectively, while the yield of peanut [14] covered with PBAT film was reduced by 1.18 % compared to PE film, without significant differences. In contrast, the yield of potato [39] covered with PBAT film was increased by 6.3%, and the yield of tomato [40] covered by PBAT film was increased by 2.4–6.0% compared to PE film. The reason for this difference might be due to different compositions of BBAT film materials, crop types, and regional environmental conditions, resulting in different effects of degradable plastic film on crop yield. The much higher yield with the PBAT and PE films compared to the CK treatment in this study indicates that film mulching is indispensable for planting crops such as tomatoes in Southern Xinjiang, particularly the Hetian region.

In terms of tomato quality, the content of tomato nutrients such as VC, soluble sugars, and soluble protein was not significantly different for each treatment due to the large variance in the same repeated groups. In contrast, Su Haiying et al. [20] showed that VC content in tomatoes was significantly higher in an open field, while soluble sugar content was significantly higher for the PABT treatment. Anwar Abduwaiti et al. [41] reported that the contents of VC and total acid in tomatoes were not significantly different for PBAT film, PE film, and an open field. These results suggest that tomato quality was not significantly affected, while the growth and yield increases were significant with PBAT film.

## 5. Conclusions

Our results showed that PBAT film started to crack and degrade after 60 days and entered its induction period. The degradation process was then accelerated, and about 60% of the PBAT film was degraded by the end of the tomato growth period, while the PE film showed no significant degradation. The effect of PBAT mulch on soil temperature and humidity was essentially equal to that of PE mulch during the seedling and flowering stages, and both were significantly higher than the effect of the CK treatment. After entering the inductive phase of the mature stage, the moisture-preserving function of the PBAT film on the soil was gradually lost due to accelerated degradation. However, this did not have any significant negative impact on tomato growth, yield, or quality. There was no significant difference in the promotion of tomato growth and yield between the PBAT and PE films, but both were significantly higher than the CK treatment, indicating that the PBAT film with an induction period of approximately 60 days essentially satisfied the growth needs of tomatoes. As a result, it is feasible to grow crops such as tomatoes in China’s southern Xinjiang region. Further studies should be focused on the feasibility of PBAT film with other crops that have different growth patterns than tomatoes and its soil safety and effects on microbial diversity and functions in the future.

## Figures and Tables

**Table 1:** The basic soil properties of the test area.

pH	Organic Matter (g/kg)	Conductivity (dS/m)	Available Nitrogen (mg/kg)	Available Phosphorus (mg/kg)	Available Potassium (mg/kg)
8.69	5.86	0.07	12.766	7.162	214.5

**Table 2:** Mass loss and degradation rate of different plastic films. The a and b symbols indicate the significance of the difference.

Type of Film Covering	Number of Days Covered with Film (d)	Final Weight (g/piece)	Weight Loss (g/piece)	Degradation Rate of Plastic Film (%)
PBAT (polybutylene adipate-co-terephthalate) film	20	0.6068a ± 0.0120	0.0132	2.13a ± 1.94
	40	0.5845a ± 0.0293	0.0355	5.73a ± 4.73
	60	0.5267a ± 0.0401	0.0933	15.05a ± 6.47
	100	0.2419b ± 0.0155	0.3781	60.98b ± 2.50
PE (polyethylene) film	20	0.5770a ± 0.0039	0.003	0.52a ± 0.63
	40	0.5754a ± 0.0039	0.003	0.79a ± 0.63
	60	0.5724a ± 0.0060	0.0076	1.31a ± 0.96
	100	0.5715a ± 0.0021	0.0085	1.47a ± 0.34

**Table 3:** Effect of different treatment methods on tomato growth. The a and b symbols indicate the significance of the difference.

	Plant Height (cm)			
**Different Treatment**	**Seedling Stage**	**Flowering and Fruit–Setting Stage**	Mature Stage	Daily Average Increment
PBAT Film	14.06a ± 1.09	35.8a ± 1.92	79.8a ± 4.76	0.858
PE Film	13.8a ± 1.96	36.8a ± 2.95	82.04a ± 5.36	0.882
CK	13.0a ± 1.58	32.6b ± 2.07	69.92b ± 5.73	0.752
	**Stem Thickness (cm)**			
**Different Treatment**	**Seedling Stage**	**Flowering and Fruit–Setting Stage**	Mature Stage	Daily Average Increment
PBAT Film	0.568a ± 0.07a	0.778a ± 0.09	1.198a ± 0.11	0.013
PE Film	0.628a ± 0.06a	0.844a ± 0.10	1.242a ± 0.10	0.013
CK	0.556a ± 0.07a	0.756a ± 0.08	1.124b ± 0.09	0.012

**Table 4:** Effect of different treatments on tomato yield. The a and b symbols indicate the significance of the difference.

Different Treatment	Average per Plant Weight/kg	% Increase/CK	% Increase/PE Film	667 m^2^ Weight/kg	% Increase/CK	% Increase/PE Film
BAT Film	1.80a ± 0.10	59.3	−9.44	3517a ± 364.6	63.38	−3.14
PE Film	1.97a ± 0.21	73.3	-	3631a ± 357.5	68.68	-
CK	1.13b ± 0.12	-	−42.6	2152b ± 237.5	-	−40.72

**Table 5:** Effect of different treatments on tomato quality. The a symbol indicates the significance of the difference.

Different Treatment	VC mg/g	Soluble Sugar (mg/g)	Soluble Protein (mg/g)
PBAT Film	0.174a ± 0.003	1.7969a ± 0.0506	0.0808a ± 0.0020
PE Film	0.272a ± 0.123	1.7890a ± 0.2775	0.0795a ± 0.0002
CK	0.287a ± 0.055	1.9310a ± 0.1495	0.0794a ± 0.0012

## Data Availability

Not applicable.
